# The Hannover Postinfarction Ventricular Septal Rupture Score: A New Scoring System Predicting 30-Day Mortality

**DOI:** 10.1016/j.cjco.2024.12.013

**Published:** 2025-01-02

**Authors:** Lisa Baustert, Dietmar Boethig, Adelheid Görler, Christian Kühn, Alexander Weymann, Bastian Schmack, Aron-Frederik Popov, Arjang Ruhparwar, Bettina Wiegmann

**Affiliations:** a Department for Cardiothoracic, Transplantation and Vascular Surgery, Hannover Medical School, Hannover, Germany; b Lower Saxony Center for Biomedical Engineering, Implant Research and Development, Hannover Medical School, Hanover, Germany; c DFG Priority Program SPP 2014, German Research Foundation, Bonn, Germany

## Abstract

**Background:**

Because of the increasing importance of quality assurance and individualised patient treatment, EuroSCOREs were analysed for reliability in predicting 30-day mortality in patients with postinfarction ventricular septal rupture (piVSR). To address the specific conditions of patients with piVSR, the Hannover piVSR Score (HpiVSR) was developed.

**Methods:**

Between 2001 and 2019, 45 patients with piVSR underwent surgery. Data were collected as necessary for EuroSCORE calculation. Clinically relevant variables were validated for the HpiVSR Score using a nonparsimonious binary logistic regression model. All models were tested for their significant predictive power for 30-day mortality. Their validity was assessed using Hosmer-Lemeshow test and Nagelkerke *R*^2^. Receiver operating characteristic curve and area under the curve were used to illustrate and quantify score accuracy.

**Results:**

The specificity was 77.8% for all EuroSCOREs and 92.6% for the HpiVSR Score, and the sensitivity was in the random range for the EuroSCOREs and 83.3% for the HpiVSR Score. Accordingly, the areas under the curve were 0.676 (95% CI 0.507-0.845) for EuroSCORE II; 0.729 (95% CI 0.581-0.878) and 0.739 (95% CI 0.591-0.886) for the additive and logistic EuroSCORE, respectively; and 0.949 (95% CI 0.891-1.006) for the HpiVSR Score.

**Conclusion:**

The HpiVSR Score enables a more reliable and accurate prediction of 30-day mortality than the EuroSCOREs using 7 significant, objective, reliable, and preoperatively determinable variables. Because of the small sample size of the present study and the fact that only internal validation has been performed so far, the weighting of the factors of the HpiVSR Score can be adjusted after studies with larger patient samples.

## Graphical abstract

**Figure:**
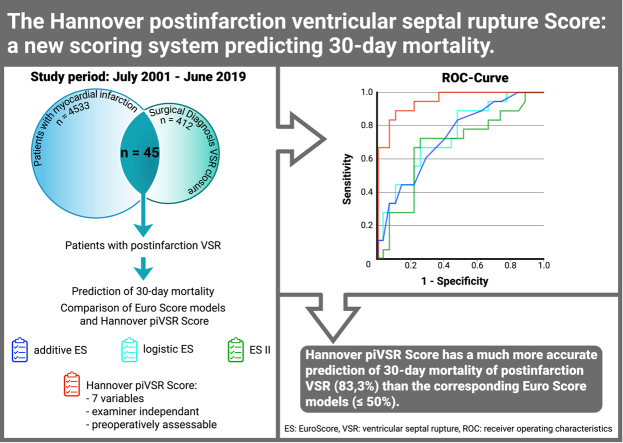

## Résumé

**Contexte:**

En raison de l'importance croissante de l'assurance qualité et du traitement personnalisé des patients, les indices EuroSCORE ont été analysés pour déterminer leur fiabilité dans la prédiction de la mortalité à 30 jours chez les patients souffrant d'une rupture du septum ventriculaire post-infarctus (RSVpi). Le score RSVpi de Hanovre (RSVpi-H) a été développé pour répondre aux conditions spécifiques des patients atteints de RSVpi.

**Méthodes:**

Entre 2001 et 2019, 45 patients atteints de RSVpi ont été opérés. Les données nécessaires au calcul des EuroSCORE ont été recueillies. Les variables pertinentes cliniquement ont été validées pour le score RSVpi-H à l'aide d'un modèle de régression logistique binaire non paramétrique. Tous les modèles ont été testés pour leur pouvoir prédictif significatif de la mortalité à 30 jours. Leur validité a été évaluée à l'aide du test de Hosmer-Lemeshow et du R^2^ de Nagelkerke. La courbe représentant la fonction d’efficacité du récepteur et l'aire sous la courbe ont été utilisées pour illustrer et quantifier la précision du score.

**Résultats:**

La spécificité était de 77,8 % pour tous les EuroSCORE et de 92,6 % pour le score RSVpi-H, et la sensibilité se situait dans la plage aléatoire pour les EuroSCORE et de 83,3 % pour le score RSVpi-H. En conséquence, les aires sous la courbe étaient de 0,676 (intervalle de confiance [IC] à 95 % 0,507-0,845) pour l'EuroSCORE II; 0,729 (IC à 95 % 0,581-0,878) et 0,739 (IC à 95 % 0,591-0,886) pour les EuroSCORE I additif et logistique, respectivement; et 0,949 (IC à 95 % 0,891-1,006) pour le score RSVpi-H.

**Conclusion:**

Le score RSVpi-H permet une prédiction plus fiable et plus précise de la mortalité à 30 jours que les EuroSCORE en utilisant 7 variables significatives, objectives, fiables et déterminables en préopératoire. En raison de la petite taille de l'échantillon de cette étude et du fait que seule une validation interne a été effectuée jusqu'à présent, la pondération des facteurs du score RSVpi-H peut être ajustée après des études portant sur des échantillons de patients plus importants.

Postinfarction ventricular septal rupture (piVSR) is a rare but severe complication of myocardial infarction (MI) that may lead to congestive heart failure with consecutive cardiogenic shock.^1^, ^2^, ^3^ Therefore, piVSR has a high 30-day mortality ranging from 40% to 61%.^4^, ^5^, ^6^ Therapeutic gold standard is surgical management, but the optimal timing to minimize perioperative morbidity and mortality remains controversial.^4^^,^^5^^,^^7^

In order to make patient-specific decisions validly, various scoring systems have been developed for cardiac surgery. They allow the prediction of 30-day mortality and the identification of high-risk patients who may benefit from initial haemodynamic stabilisation rather than immediate surgery.^8^, ^9^, ^10^ Furthermore, scoring systems enable a risk-adjusted comparison of patient outcomes between different hospitals and are used to align performance-based remuneration.^11^^,^^12^

A well-established and risk-adjusted scoring system for a broad variety of cardiac surgery patients is the "European System for Cardiac Operative Risk Evaluation" (EuroSCORE) introduced in 1999.^10^^,^^13^ This international validation study demonstrated a significant influence on postoperative mortality by 29 of the 97 studied risk factors. The resulting score represents the expected 30-day mortality after cardiac surgery and assigns patients to one of 3 risk groups. Therefore, EuroSCORE with its additive and logistic model is an assessment tool for the quality of cardiac surgery. However, studies have shown that both additive and logistic EuroSCORE overestimate postoperative mortality, with the logistic EuroSCORE even more so than the additive one.^14^^,^^15^ Therefore, in 2011, the modified EuroSCORE II was introduced in order to obtain a more contemporary estimation of hospital mortality by including cardiac and renal function as well as considering surgical urgency, new surgical standards, and improved qualities of implanted devices.^16^ This demonstrates the necessity to continuously adapt such scores and analyse their reliability and applicability to different patient cohorts and diseases.^13^

We analysed the reliability of 30-day mortality prediction in our patients with piVSR using the established EuroSCOREs. Based on our patient data, we further propose a new risk score that takes into account the time-critical management and better predicts 30-day mortality in this patient group than the EuroSCOREs.

## Methods

### Study design

After approval by the ethics committee (No. 8915_BO_K_2020), all patients who underwent surgical piVSR closure in our department between July 1, 2001, and June 30, 2019, were included. For this purpose, digital case files were searched for patients with MI (n=4533) and surgical closure of a ventricular septal defect (n = 412), of whom 45 patients were identified with true infarct-related ventricular septal rupture (VSR) after individual case review. Exclusion criteria were incomplete demographic data and associated comorbidities and peri- and postoperative data necessary for the respective EuroSCORE calculation or potentially relevant to calculate a new and more specific piVSR risk score based on literature research and own clinical experience.^2^^,^^3^^,^^6^^,^^7^^,^^17^ VSR size was collected for those patients for whom it was available. Missing VSR size, however, did not lead to the exclusion of that patient.

### Statistical analysis

For statistical analyses, we used SPSS, version 28 (IBM Corp, Armonk, NY), and MedCalc Statistical Software, version 22.009 (MedCalc Software Ltd, Ostend, Belgium). Statistical differences were considered significant if *P* was <0.05.

Factors that significantly influenced 30-day mortality in a univariable analysis were summarised for survivors and nonsurvivors in Table 1. Normally distributed continuous variables were presented as mean and standard deviation (SD), otherwise as the median and the first and third quartiles as interquartile range (IQR). Categorical variables were reported as frequencies and percentages. The significance of univariate effects on 30-day mortality for categorical variables was determined by χ^2^ test or Fisher exact test; for continuous variables, we used the independent samples *t* test (normally distributed variables as assessed by Kolmogorov-Smirnov test) or the Mann-Whitney *U* test.

**Table 1:** Demographic data and comorbidities and pre-, intra-, and postoperative data

Patient data	Total patients (N = 45)	Nonsurvivors (n = 18)	Survivors (n = 27)	*P*
Demographic data and comorbidities				
Age, y, mean ± SD	70.7 ±8.4	72.89 ±7.8	69,22 ±8.5	0.164
Men	29 (64.6)	10 (55.6)	19 (70.4)	0.309
Women	16 (35.6)	8 (44.4)	8 (29.6)	0.309
BMI, median (IQR)	27.5 (24.7-29.2)	27.6 (25.3-29.1)	26.9 (24.2-29.4)	0.444
Arterial hypertension	24 (53.3)	10 (55.6)	14 (51.9)	0.807
Diabetes mellitus	13 (28.9)	5 (27.8)	8 (29.6)	0.893
Smoking habit	9 (20)	3 (16.7)	9 (33.3)	0.215
Dyslipidaemia	9 (20)	4 (22.2)	5 (18.5)	0.761
Cerebrovascular disease	3 (6.7)	1 (5.6)	2 (7.4)	0.807
Peripheral vascular disease	4 (8.9)	2 (11.1)	2 (7.4)	0.669
COPD	3 (6.7)	0	3 (11.1)	0.264
Dialysis	2 (4.4)	2 (11.1)	0	0.076
NYHA state				0.350
I	0	0	0	
II	11 (24.4)	5 (27.8)	6 (22.2)	
III	12 (26.7)	3 (16.7)	9 (33.3)	
IV	17 (37.8)	9 (50)	8 (29.6)	
Unknown	5 (11.1)	1 (5.6)	4 (14.8)	
Coronary artery disease				0.705
1-vessel	16 (35.5)	6 (33.3)	10 (37.0)	
2-vessel	12 (26.6)	6 (33.3)	6 (22.2)	
3-vessel	17 (37.8)	6 (33.3)	11 (40.7)	
Preoperative data				
Time MI to VSR, d, median (IQR)	4 (1-7.5)	3 (1-6.25)	5 (3-8)	0.063
Time VSR to surgery, d, median (IQR)	1 (0-5.5)	0 (0-1)	3 (0-13)	<0.001
Critical preoperative status∗	26 (57.8)	14 (77.8)	12 (44.4)	0.027
LVEF <45%	29 (69.0)	13 (76.5)	16 (64.0)	0.305
Preoperative mechanical support	15 (33.3)	6 (33.3)	9 (33.3)	>0.999
IABP	10 (22.2)	5 (27.8)	5 (18.5)	0.489
ECMO	4 (8.9)	1 (5.6)	3 (11.1)	0.640
Impella + ECMO	1 (2.2)	0	1 (3.7)	0.600
Intraoperative data				
Concomitant operation	34 (75.6)	13 (72.2)	21 (77.8)	0.671
CABG	34 (75.6)	13 (72.2)	21 (77.8)	0.671
Valve	5 (11.1)	2 (11.1)	3 (11.1)	>0.999
Total operation time, min, mean ± SD	246.8 ±65.6	266.6 ±66.8	233.6 ±62.6	0.100
Cardiopulmonary bypass time, min, mean ± SD	166.2 ±60.2	193.8 ±64.6	147.8 ±50.2	0.010
Aortic cross-clamp time (min), median (IQR)	78 (61.0-100.50)	76.5 (63.75-100)	78 (51-102)	0.917
VSR size (cm^2^), median (IQR)†	2 (1.125-2.0) ^a^	2 (1-2.38)	2 (1.5-2)	0.954
Intraoperative mortality	5 (11.1)	5 (27.7)	0	/

	Total patients (n = 40)	Nonsurvivors (n = 13)	Survivors (n = 27)	*P*

Postoperative data				
Postoperative mechanical support	26 (65.0)	11 (84.6)	15 (55.6)	0.090
ECMO	8 (20.0)	4 (30.8)	4 (14.8)	0.400
IABP	16 (40.0)	6 (46.2)	10 (37.0)	0.581
ECMO + Impella	1 (2.5)	0	1 (3.7)	>0.999
ECMO + Impella + IABP	1 (2.5)	1 (7.7)	0	0.325
No ventilation	18 (40.0)	7 (38.9)	11 (40.7)	0.901
Residual VSR	6 (15.0)	2 (11.1)	4 (18.2)	0.673
Revision surgery	8 (17.8)	4 (30.8)	4 (14.8)	0.400
Stay in intensive care unit, d, median (IQR)	5 (1.0-13.0)	1.5 (0-9.25)	6 (3-16)	0.121
Total hospital stay, d, median (IQR)	13 (3.5-19.5)	1.5 (0-9.25)	15 (13-26)	<0.001
Postoperative mortality	13 (32.5)	13 (100)	0	/

Unless otherwise noted, data are expressed as number and percentage of, respectively, total patients, nonsurvivors, or survivors.

BMI, body mass index; CABG, coronary artery bypass graft; COPD, chronic obstructive pulmonary disease; ECMO, extracorporeal membrane oxygenation; IABP, intra-aortic balloon pump; IQR, interquartile range; LVEF, left ventricular ejection fraction; MI, myocardial infarction; NYHA, New York Heart Association; SD, standard deviation; pts, patients; VSR, ventricular septal rupture.

∗ At least 1 of the factors: inotropic support, preoperative resuscitation for ventricular tachycardia/fibrillation or cardiac arrest.

† Available only for 40 patients.

The additive and logistic EuroSCORE as well as the EuroSCORE II were calculated using the online calculator (https://euroscore.org/calcold.html) of the "British Medical Journal" and the "Patient's Internet Handbook" (Table 2).

**Table 2:** Calculating additive, logistic EuroSCORE and EuroSCORE II

EuroSCORE	Total patients (N = 45)	Nonsurvivors (n = 18)	Survivors (n = 27)	*P*
Additive EuroSCORE, mean ± SD	15.9 ± 4.2	17.9 ± 3.9	14.5 ± 3.8	0.005
Logistic EuroSCORE, %, mean ± SD	58.2 ± 25.4	70.7 ± 21.0	49.9 ± 25.0	0.006
EuroSCORE II, %, median (IQR)	17.8 (6.6-34.7)	30.6 (10.4-45.5)	12.2 (5.3-19.4)	0.048

IQR, interquartile range; SD, standard deviation.

We included any studied risk factors that showed significant influence on 30-day mortality in patients with piVSR in our proposed Hannover piVSR (HpiVSR) Score. Through our literature search, we identified other well-established risk factors that did not show a significant influence in our study because of the limited sample size. However, if these risk factors were deemed investigator-independent and relevant within the limited time frame of piVSR management, we included them in our score. This resulted in the following variables: age (years), gender (male, female), time from MI diagnosis to the diagnosis of VSR (days), time from VSR diagnosis to surgery (days), critical preoperative status (at least 1 of the factors: inotropic support, preoperative resuscitation for ventricular tachycardia/fibrillation or cardiac arrest), preoperative extracorporeal membrane oxygenation (ECMO), or intra-aortic balloon pump (IABP) and concomitant surgery (coronary artery bypass graft [CABG], valve replacement). Through a regression model, we then calculated the weighting of the individual variables according to the endpoint "30-day mortality."

**Figure 2:**
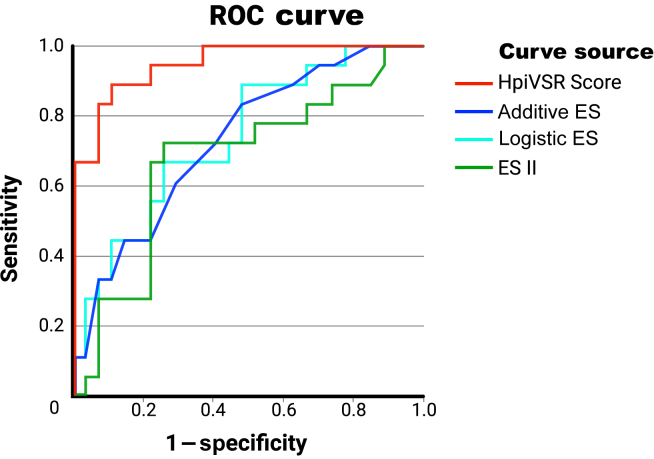
ROC curves of the different scoring models. ES, EuroSCORE; HpiVSR, Hannover Postinfarction Ventricular Septal Rupture; ROC, receiver operating characteristic.

The selected regression model was then tested with the omnibus test of model coefficients to determine whether it significantly predicted the probability of 30-day mortality. Classification tables were used to check prediction behaviour. Hosmer-Lemeshow test and Nagelkerke *R*^2^ were used to test goodness of fit. The goodness of fit for Hosmer-Lemeshow is assumed if differences between the subgroups formed by repeated omission of a sample fraction remain nonsignificant. The model’s discriminatory power was assessed using the receiver operating characteristic (ROC) curve (Figure 2). To quantify the discriminatory power, the area under the curve (AUC)—with an AUC value of 0.5 indicating a lack of it and a value closer to 0 or 1 indicating better goodness of fit—was used. Finally, the AUC of the respective EuroSCORE was compared directly with the HpiVSR Score (Table 5).

**Table 5:** Results of the scoring model comparisons

Result	Additive EuroSCORE	Logistic EuroSCORE	EuroSCORE II	HpiVSR Score
Prediction survival correct, specificity, %	77.8	77.8	77.8	92.6
Prediction death correct, sensitivity, %	44.4	50.0	33.3	83.3
Diagnostic accuracy, %	64.4	66.7	60.0	88.9
Positive predictive value, %	57	60	50	88
Negative predictive value, %	68	70	64	89
Nagelkerke *R*^2^	0.222	0.220	0.119	0.756
AUC (95% CI)	0.729 (0.581-0.878)	0.739 (0.591-0.886)	0.676 (0.507-0.845)	0.949 (0.891-1.006)
Hosmer-Lemeshow test	χ^2^(8) = 2.5 *P* = 0.959	χ^2^(7) = 4.5 *P* = 0.715	χ^2^(7) = 5.3 *P* = 0.622	χ^2^(7) = 2.415 *P* = 0.933
Significance of pairwise AUC comparison to HpiVSR Score, *P*	0.003	0.005	0.001	—

AUC, area under the curve; HpiVSR, Hannover Postinfarction Ventricular Septal Rupture; CI, confidence interval.

## Results

### Patient analysis

Demographic data and comorbidities, perioperative data, and postoperative data are listed in Table 1. The mean age was 70.7 ± 8.4 years, with the majority of patients being male (64.6%). There was a significant time difference between diagnosis and surgery for survivors and nonsurvivors. Nonsurvivors had surgery within 0 (0-1) days of diagnosis, with 72.2% undergoing surgery less than 24 hours after diagnosis. The median for survivors was 3 (3-13) days (*P* < 0.001), with 11 patients (24.2%) undergoing surgery after >7 days.

Overall, 57.8% of patients had a critical preoperative status, which was significantly higher in nonsurvivors (77.8%) compared with survivors (44.4%, *P* = 0.027). Finally, the mean cardiopulmonary bypass time was significantly higher in nonsurvivors (193.8 ± 64.6 minutes vs 147.8 ± 50.2 minutes, *P* = 0.010). VSR size was available for only 40 patients.

Postoperative data collection (Table 1) was performed on 40 patients (5 died intraoperatively). Mean duration of intensive care stay was comparable in both groups, but the mean total length of stay was significantly higher in survivors at 15 days (13-26) and can be explained by the postoperative mortality of 32.5% (n=13). The 30-day mortality of the overall cohort was 40% (n = 18). The 30-day mortality rate changed over the study period from 50% (13 nonsurvivors among 26 patients) between 2001 and 2009 to 26% (5 nonsurvivors among 19 patients) between 2010 and 2019.

### EuroSCOREs

The calculation of the additive EuroSCORE showed a mean value of 15.9 ±4.2 for the entire cohort. At 17.9 ± 3.9, the mean value in nonsurvivors was significantly higher than that of survivors (14.5 ± 3.8; *P* = 0.005). According to the classification of Nashef et al.,^10^ all 45 patients were assigned to the highest risk category. The calculation of the logistic EuroSCORE resulted in a mean value of 58.2 ± 25.4 for the entire patient cohort, which is equivalent to the percentage probability of 30-day mortality. Mean values of the subgroups also differed significantly (nonsurvivors vs survivors: 70.7 ± 21.0 vs 49.9 ± 25.0, *P* = 0.006).

EuroSCORE II values are reported as median and IQR, which were 17.8 (6.6-34.7) for the total cohort. It also showed significant differences between the 2 subpopulations, with 30.6 (10.4-45.5) for nonsurvivors vs 12.2 (5.3-19.4) for survivors (*P* = 0.048) and corresponded to the percentage probability of 30-day mortality. In summary, the predicted mortality differed significantly between survivors and nonsurvivors in all 3 EuroSCORE models (Table 2).

### HpiVSR Score

According to the previously established criteria, following variables were included in the calculation of the HpiVSR Score: age, gender, time from MI diagnosis to the diagnosis of VSR, time from VSR diagnosis to surgery, critical preoperative condition, preoperative mechanical support, and concomitant surgery. These variables were entered into a Cox binary regression model with the corresponding endpoint of 30-day mortality. Factor weights and associated statistical information are presented in Table 3, which shows an increased risk of 30-day mortality for older patients and critical preoperative status with a positive regression coefficient or odds ratio >1. A favourable influence on 30-day mortality was shown for men, a longer time between MI and VSR, a longer time between VSR and surgery, preoperative mechanical support, and concomitant surgery, which have a negative regression coefficient or odds ratio <1.

**Table 3:** HpiVSR Score: defined variables and regression coefficients for calculation

Variables	Regression coefficient	Standard error	Significance	Odds ratio	95% CI for odds ratio
Age, y	0.215	0.106	0.043	1.240	1.007-1.526
Gender (male: 1, female: 0)	–2.479	1.655	0.134	0.084	0.003-2.149
Time MI to VSR, d	–0.371	0.203	0.068	0.690	0.463-1.027
Time VSR to surgery, d	–2.169	0.927	0.019	0.114	0.019-0.703
Critical preoperative state	0.954	1.306	0.465	2.596	0.201-33.604
Preoperative mechanical support	–1.772	1.352	0.190	0.170	0.012-2.403
Concomitant surgery	–1.283	1.426	0.368	0.277	0.017-4.532
Constant	–8.964	6.800	0.187	0.000	

CI, confidence interval; HpiVSR, Hannover Postinfarction Ventricular Septal Rupture; MI, myocardial infarction; VSR, ventricular septal rupture.

To calculate the individual HpiVSR Score for each patient, 2 steps are required. First, the sum of the constant and the products of the factor values multiplied by their regression coefficients is calculated.

Step 1:

SUM=−8.964+(Age[years]×0.215)+(Gender[female=0,male=1]×−2.479)+(TimeMItoVSR[days]×−0.371)+(TimeVSRtosurgery[days]×−2.169)+(Criticalpreoperativestate[no=0,yes=1]×0.954)+(Preoperativemechanicalsupport[no=0,yes=1]×−1.772)+(Concomitantsurgery[no=0,yes=1]×−1.283)

This result (SUM) is then inserted into the following formula to obtain the HpiVSR Score.

Step 2:

$$\text{HpiVSR}\;\text{Score}=\frac{1}{\left(1+e^{-\text{SUM}}\right)}$$

HpiVSR Score has values between 0 and 1. Multiplying by 100 gives the estimated percentage of 30-day mortality after piVSR surgery, which was 25.9% for the total cohort, 90.2% for nonsurvivors, and 1.7% for survivors (Table 4). Omnibus test of the model coefficients showed significance for the entire model (*P* < 0.001).

**Table 4:** Calculated HpiVSR Score for the total cohort, as well as the subgroups of nonsurvivors and survivors

Measure	Total patients (N = 45)	Nonsurvivors (n = 18)	Survivors (n = 27)	*P*
HpiVSR Score, median (IQR)	25.9 (0.0-87.2)	90.2 (66.1-97.7)	1.7 (0.0-23.3)	<0.001

HpiVSR, Hannover Postinfarction Ventricular Septal Rupture; IQR, interquartile range.

**Figure 1:**
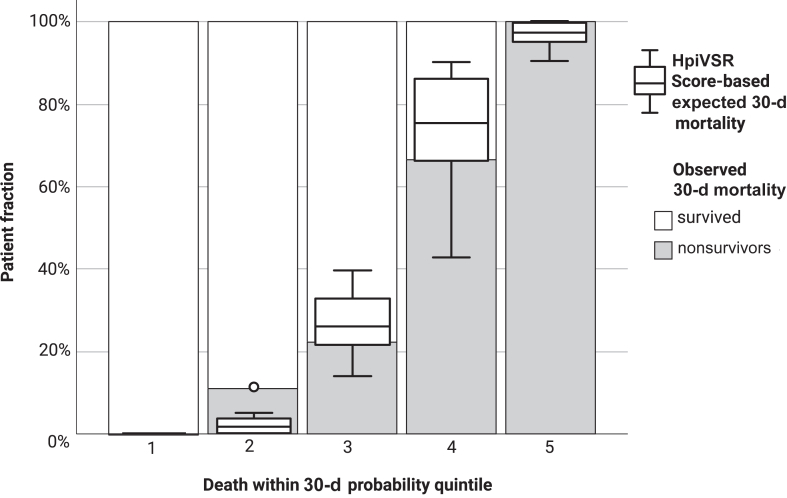
Plot of observed and expected probabilities of death in 5 risk quintiles. Medians and interquartile ranges for score-predicted survival are also shown. HpiVSR, Hannover Postinfarction Ventricular Septal Rupture.

Figure 1 shows the quintiles of survival probability on the *x* axis and the observed survivors and nonsurvivors as stacked bars above. It shows that the score is able to distinguish well between patients in 5 groups, with increasing risk and steadily increasing mortality.

### Comparisons of model performance

Table 5 shows that the correctly predicted survival rate (specificity) of 77.8% is identical for all 3 EuroSCOREs, whereas the HpiVSR Score has a correctly predicted survival rate of 92.6%. The correct prediction of death (sensitivity) for all 3 EuroSCOREs is in the range of or worse than random, with the logistic EuroSCORE having the best predictive power at 50%. In comparison, the value for the HpiVSR Score is 83.3%. The overall correct prediction was 64.4% for the additive, 66.7% for the logistic, and 60.0% for the EuroSCORE II. The HpiVSR Score has a significantly better predictive power of 88.9% (Table 5).

The goodness of fit was assessed by Nagelkerke *R*^2^ and, according to the interpretation of Backhaus et al.,^18^ shows a lack of effect for the EuroSCORE II with an *R*^2^ <0.2, a small, acceptable effect for the additive and logistic EuroSCORE (*R*^2^ = 0.222, *R* = 0.220) and a large effect for the HpiVSR Score with *R*^2^=0.756. In addition, the goodness of fit was calculated using the Hosmer-Lemeshow test, which showed no significant differences between the subgroups for all scores, which can therefore be regarded as internally validated.

The discriminatory power of the individual tests was assessed using AUC, with the EuroSCORE II having the lowest value of 0.676 ± 0.085, followed by 0.729 ± 0.075 for the additive and 0.739 ± 0.075 for the logistic EuroSCORE. An AUC of 0.949 ± 0.030 for the HpiVSR Score indicates very good discriminatory power. In a direct comparison of AUC values, the HpiVSR Score performed significantly better than any of the EuroSCORE models (Table 5). The quality of the models was assessed using the ROC curve (Figure 2).

## Discussion

### Principal findings

The HpiVSR Score enables a more reliable and accurate prediction of 30-day mortality in our piVSR patient cohort than additive and logistic EuroSCORE or EuroSCORE II. All 7 variables required for the calculation of the HpiVSR Score are investigator-independent and relevant within the limited time frame of piVSR management.

### Patient analysis

Overall, the incidence of piVSR was relatively high at 1% compared with the current literature at around 0.2% maybe because of the supraregional referral of patients from other hospitals (9 of 45 patients).^3^^,^^19^^,^^20^ This leads to high expertise in pre-, intra-, and postoperative patient care, which explains a comparatively low 30-day mortality rate of 40%.

Demographic distribution of the patient population, with a predominantly male population and a mean age of 70.7 years, is consistent with the current literature, in particular the review by Matteucci et al.,^7^ which found a mean age of 67.1 ± 4.3 years and 55.4% male patients.^21^ Overall, our cohort showed a similar cardiovascular risk profile as in other studies, in particular arterial hypertension and diabetes mellitus.^5^^,^^6^^,^^19^ As already described by others, multivessel disease was not found to be a significant factor in our study.^17^^,^^20^ These findings suggest that our cohort is a suitable sample for the entirety of patients with piVSR.

The significant influence of cardiopulmonary bypass time on 30-day mortality described in other studies was also confirmed for this patient population.^6^ This implies the necessity for individual risk-reward consideration between either a prolonged operating time or a 2-stage surgical procedure with VSR closure and temporary mechanical circulatory support before CABG.^6^ However Kutty et al.^22^ describe the negative effects of incomplete myocardial revascularisation on long-term survival. This emphasizes the need for a surgical team with high expertise in dealing with this particular group of patients.

### Scoring systems

A scoring system can be an instrument for the surgeon to decide whether the patient should be operated immediately or would benefit from an initial stabilisation with intensive care unit stay or mechanical support systems. For patients with piVSR, this is a controversial question in the literature.

Compared with other studies, the subset of nonsurvivors in our cohort had a significantly shorter time between piVSR diagnosis and surgery (0 vs 3 days), which was probably due to haemodynamic instability, as a critical preoperative state had a significant impact on outcome (*P* = 0.027).^6^^,^^17^^,^^23^ Ariza-Solé et al.^1^ found a comparable intrahospital mortality rate in patients if those with a higher risk profile had undergone stabilisation with mechanical support systems first. Poulsen et al.^5^ also identified sudden haemodynamic instability as a risk factor for poor surgical outcomes with higher mortality rates, proposing that haemodynamic stabilisation using IABP or ECMO should be performed first. Only if this is not feasible, immediate surgery is recommended.^24^ Other authors also reported better outcomes with mechanical support systems in haemodynamically unstable patients.^1^^,^^25^

Although a recommendation for clinical decision making regarding treatment options is beyond the scope of this study, a better and validated risk score is a prerequisite for further studies regarding this question. Furthermore, the scoring system is intended to help assess, measure, and compare the quality of cardiac surgery outcomes.^10^

#### EuroSCOREs

In summary, the results of the 3 EuroSCOREs for our cohort differ significantly. Their predictions of 30-day mortality of our surviving and nonsurviving patients with piVSR are in the range of random selection and thus not suitable. Furthermore, they only partly fulfil the necessary criteria for complex and time-critical management in patients with piVSR, as they are determined by a large number of variables that require the cooperation of awake, neurologically oriented patients (eg, medical anamnesis), are partially observer-dependent (eg, determination of left ventricular ejection fraction) and require the additional use of diagnostic methods (eg, quantification of pulmonary arterial hypertension).^10^^,^^13^^,^^16^ In addition, the EuroSCORE models were validated on the basis of previous cardiac surgery in all adult patients. The aspect of high-risk patients, as is the case with piVSR, was not specifically taken into account. Guillet et al.^26^ already assessed the EuroSCORE II as inadequate for the assessment of high-risk patients.

#### Hannover piVSR Score

Because of the lack of reliability of the EuroSCOREs regarding the valid prediction of 30-day mortality in patients with piVSR, it became necessary to propose an alternative score. The HpiVSR Score is composed of 7 investigator-independent, reliable, fast, and simple variables that can be determined preoperatively without excessive additional diagnostic effort. As previously described by other authors, we included both age and gender as significant risk factors in the calculation of the score, as they influence both the occurrence of piVSR and 30-day mortality.^2^^,^^6^^,^^19^ In this cohort, for example, older age and female gender in the multivariable analysis was associated with higher 30-day mortality. Other variables that significantly reduced mortality were the time between MI and VSR as well as VSR and surgery. These have also been described as important parameters by other authors.^3^^,^^17^^,^^20^^,^^23^

Analysis of our preoperative data revealed a median time of 4 days between MI and piVSR diagnosis, which was 3 days in the nonsurvivor group and 5 days in the survivor group. This difference may be due to the timing of diagnosis not coinciding with the onset of piVSR; that is, milder symptoms in the survivors may have led to a delayed diagnosis, especially considering that 9 patients were referred from peripheral hospitals where this diagnosis is more likely to be missed because of its low incidence.

In order to enable comparability regardless of the availability of the various mechanical support systems and the small number of cases, the parameter was generalised and not separated according to the individual systems. Historically, IABP was favoured over ECMO until around 2013, particularly because of better experience and greater availability.^2^^,^^3^^,^^5^^,^^17^^,^^24^ This is also reflected in our data, in which the first use of ECMO was in 2014 and which subsequently replaced the IABP as the standard of care. The potential advantages of ECMO, such as systemic circulatory support and easier weaning from cardiopulmonary bypass, should be analysed in more detail in the future in order to make a recommendation for optimal mechanical circulatory support in patients with piVSR.^1^^,^^27^^,^^28^

Labrousse et al.^29^ already found that concomitant CABG was associated with a lower risk of in-hospital mortality as did we. The authors described that it was beneficial in particular to reduce the risk of subsequent ischaemia and to improve the chance of myocardial healing. Furthermore, incomplete revascularisation seems to have a negative impact on long-term survival.^26^ In contrast, a critical preoperative condition, defined by preoperative inotropic medication or resuscitation, increased the probability of 30-day mortality significantly.^6^^,^^30^

It may seem obvious that VSR size has an influence on outcome. In our cohort, however, VSR size was only determined in 9 patients by preoperative echo. In another 8 patients, haemodynamic relevance but not VSR size was determined. Subsequent analysis revealed no significant difference in VSR size between survivors and nonsurvivors in relation to 30-day mortality. This finding is confirmed in a study by Helmcke et al.^31^ In addition to the lack of significance, determination of VSR size is observer-dependent and is rarely performed preoperatively, which in any case only reflects a snapshot and would therefore have to be performed immediately before surgery. Because of these many limitations, VSR size was not included in the HpiVSR Score.

Because of the unstable clinical condition of these patients, continuous evaluation using the Hannover piVSR Score is both appropriate and necessary in order to quickly recognize changes in the patient's condition and act accordingly.

### Strengths and limitations

When predicting piVSR mortality, the specific HpiVSR Score performed significantly better than the previous models. Both the quality of the model and the discriminatory power were superior to the more general EuroSCOREs. Limitations exist because of the small sample size. As a result of the low incidence, there are many publications of individual case reports or small sample size and rarely studies with larger numbers of cases.^2^^,^^5^^,^^7^^,^^15^ In order to achieve an initially sufficient cohort of 45 patients at our cardiac centre, which is comparably large for a single-centre analysis, we have chosen a study period of 18 years.^6^^,^^32^^,^^33^ However, this has the disadvantage that we cannot exclude possible influences of improvements in surgical care, medical technology, and general patient care over this long study period (eg, the improvement in clinical experience and the use of ECMO). These improvements may be reflected in the change in 30-day mortality over the study period and the reduction in intraoperative mortality. In addition, all intraoperative deaths occurred within the first third of the study period, before 2007, which were not taken into account in the analysis.

Some nonsignificant factors (age, sex, time MI to VSR, preoperative mechanical support, and concomitant surgery) were included in our model as they have already been shown to be significant in other studies, so we would expect them to be significant in a larger patient population.^2^^,^^3^^,^^6^^,^^7^^,^^15^ Owing to the observational nature of the study, the association between the longer time from VSR and surgery and a better survival profile could potentially be influenced by other risk factors such as haemodynamic instability.

Another limitation is the possibility of a so-called immortal time bias. This was investigated by González-Pacheco et al.^34^ and describes a statistically incorrect conclusion regarding the survival benefit of a therapy. In this study, we only looked at patients who underwent surgery and none who received drug therapy. This could lead to an incorrect conclusion for patients who did not undergo surgery. However, as surgery is considered the gold standard in the general literature and we have assumed planned surgery as a prerequisite, we consider this to be negligible in our study.^4^^,^^5^^,^^7^ Nevertheless, we understand the authors’ concerns, and future studies should address this phenomenon and the potential impact on the standard of care.

A major limitation of the study is its retrospective nature and the lack of external validation. We applied the score to the developmental population only and consider it as a suggestion that needs to be externally validated in a larger multicenter patient cohort. Because of the small sample size of the present study and the fact that only internal validation has been performed so far, the weighting of the factors of the HpiVSR score can be adjusted based on studies with larger patient samples.

## Conclusions

The HpiVSR Score predicts 30-day mortality after surgical treatment of septal rupture following a MI. It comprises 7 factors that are easy to determine preoperatively: age, gender, time from MI diagnosis to the diagnosis of VSR, time from VSR diagnosis to surgery, critical preoperative status, preoperative mechanical support, and concomitant surgery. Prediction abilities in the piVSR population are promising and much more accurate than the predictions of the EuroSCORE family. Validation with data from other institutions is needed.
